# Redox‐Active Tungsten Mono‐Oxo Bis(dithiolene) Complex: A Fast‐Rechargeable Anode for High‐Capacity Lithium‐Ion Batteries

**DOI:** 10.1002/advs.75010

**Published:** 2026-03-25

**Authors:** Honggyu Seong, Jaeheon Lee, Jae Hyun Park, Woonghee Lee, Junhyeok Seo, Jaewon Choi

**Affiliations:** ^1^ Department of Chemistry Gyeongsang National University Jinju Republic of Korea; ^2^ Gyeongnam Aerospace & Defense Institute of Science and Technology Gyeongsang National University Jinju Republic of Korea; ^3^ Department of Chemistry Gwangju Institute of Science and Technology Gwangju Republic of Korea; ^4^ Research Center for Innovative Energy and Carbon Optimized Synthesis for Chemicals (Inn‐ECOSysChem) Gwangju Institute of Science and Technology Gwangju Republic of Korea; ^5^ School of Aerospace Engineering Gyeongsang National University Jinju Republic of Korea; ^6^ Department of Chemistry University of Colorado Denver Denver Colorado USA

**Keywords:** anode materials, fast rechargeabilities, lithium‐ion batteries, redox‐active metal complexes, tungsten mono‐oxo bis(dithiolene) complexes

## Abstract

The increase in electric vehicle popularity has heightened the need for high‐energy lithium‐ion batteries (LIBs). LIB performance has been steadily improved, but next‐generation energy storage systems have yet to be developed. However, redox‐active organometallic complexes have unique electrical properties with the potential for integration into LIB systems as promising electrode materials. Herein, the Li‐ion storage capability of a redox‐active W‐oxo bis(dithiolene) complex is explored as a novel anode material for LIBs. Based on these redox properties, the W‐oxo bis(dithiolene) complex anode demonstrated electrochemical reversibility in lithiation‐delithiation processes and outstanding rate performance (698 mAh g^−1^ after 1000 cycles at 10 A g^−1^). Ex situ X‐ray photoelectron spectroscopy analysis, computational simulations, and further electrochemical investigations were performed to investigate the electrochemical Li‐ion storage mechanism of the W‐oxo bis(dithiolene) complex anode. This work presents a new strategy for high‐capacity LIBs by incorporating a redox‐active W‐oxo bis(dithiolene) complex into an unprecedented anode material.

## Graphical abstract

This study reports the first application of a W‐oxo bis(dithiolene) complex, (Et_4_N)_2_[WO(S_2_C_2_(C_6_H_5_)_2_)_2_], as a novel anode material for lithium‐ion batteries. This complex stores Li^+^ ions via multivalent W‐centered redox processes involving its oxo (WO) and dithiolene (S) sites. The W‐complex anode delivers high reversible capacity and outstanding rate performance up to 10.0 A g^−1^, enabling fast‐charge energy storage.

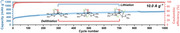

## 1 Introduction

Growing demands for grid‐scale energy storage systems have driven lithium‐ion batteries (LIBs) toward higher energy density. Furthermore, the miniaturization of electrical devices and the rapid expansion of electric vehicles have urgently required LIBs to possess high energy density, fast rechargeability, and long cycle lifetimes. LIB performance is largely determined by the electrode materials, which define key parameters, including specific capacity, operating voltage, coulombic efficiency, and rate capability. However, widely used materials, such as LiNi_x_Co_y_Mn_z_O_2_ (NMC) cathodes and graphite anodes, are constrained by intrinsic limitations. Their poor rate capabilities and relatively low specific capacities (e.g., LiNi_0.8_Co_0.1_Mn_0.1_O_2_: 215 mAh g^−1^ and LiC_6_: 372 mAh g^−1^) hinder the production of high‐performance LIBs [1, 2]. Therefore, new electrode materials that can deliver superior cycling stability and higher energy density must be identified and developed to improve LIB performance.

Electrode materials for LIBs must exhibit sufficient redox activity, as they store and release Li^+^ ions through electrochemical redox reactions during charge‐discharge processes. In this regard, organometallic compounds are promising LIB electrode materials because they have exhibited unique electrochemical properties in various fields of electrochemistry [3, 4, 5]. Organometallic complexes can have more divergent electrochemical tunability than organic compounds by controlling not only the chemical structures of their organic ligand component but also coordinated transition metal centers [6]. Altering the chemical structures of the ligand component and the species of the coordinated metal center changes the electronic environment of these metal complexes. Moreover, multi‐electron redox processes in both the metal centers and ligand components enable them to function as high‐capacity novel electrode materials. This approach can considerably alter the electrochemical properties of organometallic complexes, which are directly related to LIB performance parameters that affect energy density, including working voltage and specific capacity. Thus, exploring various organometallic compounds can achieve broader insight into their unique electrochemical properties in LIB systems. Along these lines, coordination compounds have recently been investigated as alternative anode materials for LIBs, where both metal centers and ligands can participate in multi‐electron redox. For instance, pincer‐type Co(II) and Zn(II) chloride complexes have been reported to exhibit high reversible capacities, which were ascribed to a conversion reaction involving LiCl formation accompanied by metallic Li deposition [7]. In addition, various coordination‐based anode materials such as Cu complexes [8, 9] and coordination polymers [10] have also shown competitive lithium storage performance. Furthermore, coordination‐motif‐based interfacial designs have also been reported in lithium metal battery systems. For example, Ni‐bis(dithiolene) motifs have been used as lithiophilic interfacial sites to regulate Li nucleation and deposition. These motifs were introduced either as a self‐assembled interfacial layer on Li metal anode [11] or as Ni‐bis(dithiolene)‐containing COF guiding layers [12], resulting in more uniform Li deposition and lower polarization. Although these studies focus on Li metal anodes, they highlight that sulfur‐rich dithiolate coordination environments can influence Li‐related interfacial processes.

Dithiolene is a redox non‐innocent ligand that exhibits diverse oxidation/reduction reactivity while coordinating with transition metal ions [13], particularly stabilizing high‐valent W‐oxo complexes [14]. In the axial position of the dithiolene‐W coordination environment, the W‐oxide moiety becomes highly nucleophilic [15]. The sulfur sites on the dithiolate ligand also exhibit enhanced nucleophilicity due to localized electron density, enabling both W‐oxo and dithiolene‐S sites to interact with alkaline metal ions or protons reversibly [16, 17, 18]. A recent study reported hydrogen‐bonding interactions between proton donors and both the oxo and dithiolene‐S sites in W‐oxo bis(dithiolene) complexes, demonstrating its capability to store electrical energy as H‐H chemical bonds through proton reduction [15].

These findings further suggest the potential applicability of such complexes as anode materials for energy storage involving Li^+^ ions. Notably, the reactivity of these complexes with Li^+^ cations and their electron transfer properties are critical factors determining their effectiveness as anode materials. Electron transfer to the low‐spin W^IV^ d orbitals in bis(dithiolene) W‐oxo complexes is challenging due to the significant HOMO‐LUMO energy gap [14]. Additionally, utilizing the π* orbital of dithiolate requires substantial potential, theoretically −3.45 V versus Fc^+/0^ [15]. However, previous studies reported that electron transfer can occur at considerably lower energy levels via the molecular orbital rearrangements induced by proton‐coupled electron transfer, suggesting that electron transfer facilitated by Li^+^ cations could also be feasible [15].

In this context, this work investigated the potential of the W‐oxo bis(dithiolene) complex as a novel anode material for LIBs. This concept was successfully applied in a lithium‐ion half‐cell system and showed excellent rate capabilities with a high reversible capacity of 698 mAh g^−1^ over 1000 cycles in galvanostatic charge‐discharge cycling tests at a high current density of 10 A g^−1^. This study introduces the fast‐rechargeable ion storage mechanisms of the W‐oxo bis(dithiolene) complex anode via tungsten and ligand‐based redox processes with capacitive electrochemical behaviors, as demonstrated using ex situ X‐ray photoelectron spectroscopy (XPS) analysis, density functional theory (DFT)‐based calculations, and various electrochemical measurements. The study and its findings are expected to provide a foundation for the application of both tungsten complexes and other dithiolene‐based metal complexes as next‐generation LIB anode materials.

## 2 Results and Discussion

In this study, a W complex capable of stable electrochemical oxidation/reduction reactions, namely (Et_4_N)_2_[WO(pdt)_2_] ((Et_4_N)_2_[**1**], pdt = S_2_C_2_(C_6_H_5_)_2_), was utilized to investigate whether a high‐valent W‐oxo complex could function as an anode material in LIBs. (Et_4_N)_2_[**1**] was synthesized according to reported methods [19], and the corresponding one‐electron oxidized state was also prepared [19]. Initially, the reactivity of the W═O species toward Li^+^ cations was assessed by observing changes in the v(W═O) stretching frequency under LiPF_6_ conditions. As expected, the interactions between W═O and Li^+^ shifted the v(W═O) toward lower frequencies.

**FIGURE 1:**
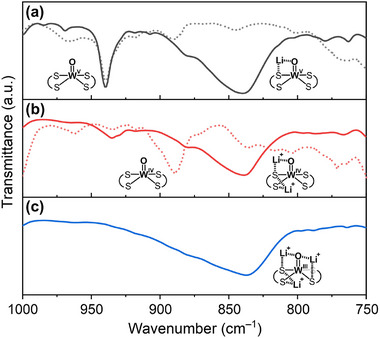
FT‐IR spectra of (a) [**1**]^1−^ with 1 equivalent of LiPF_6_, showing a v(W^V^═O) shift, (b) [**1**]^2−^ with 2 equivalents of LiPF_6_, showing a v(W^IV^═O) shift, and (c) after reducing [**1**]^2−^ using 1 equivalent of Na(Hg) in the presence of 3 equivalents of LiPF_6_.

A reaction of (Et_4_N)[**1**] with 1 equivalent of LiPF_6_ resulted in a peak shift from 940 to 840 cm^−1^ regarding the v(W^V^═O) stretching frequency (Figure 1a). These changes indicate that Li^+^ cation interactions reduce the electron density on the W═O bond, making the W center more electron‐deficient and facilitating reduction reactions. Although a significant peak shift was observed, the original v(W═O) peak remained, with the intensity ratio between the original and shifted peaks (1:2.4). This observation suggests that the W^V^═O─Li^+^ state is thermodynamically favored but exists in equilibrium with the W^V^═O state, consistent with a small free energy difference.

Subsequently, changes in v(W^IV^═O) were also examined in the presence of LiPF_6_. The reaction of (Et_4_N)_2_[**1**] with 2 equivalents of LiPF_6_ caused a v(W^IV^═O) peak shift from 890 to 839 cm^−1^ (Figure 1b). Further reduction of the W^IV^═O state to W^III^═O was enabled in the presence of LiPF_6_. An in situ experiment including (Et_4_N)_2_[**1**] with 1 equivalent of Na(Hg) under 3 equivalents of LiPF_6_ resulted in the formation of W^III^═O species at 837 cm^−1^ (Figure 1c).

The effect of interactions between Li^+^ cations and W complex upon electron transfer was investigated under electrochemical conditions. (Et_4_N)_2_[**1**] exhibited redox couples corresponding to the W^V‐IV^ reduction/oxidation at −1.06 V versus Fc^+/0^ (Figure S1). Cyclic voltammograms obtained under LiPF_6_ conditions showed that (Et_4_N)_2_[**1**] displayed a positive shift in the W^V‐IV^ reduction potential by 50 mV (to −1.01 V vs Fc^+/0^) and a new reduction peak at −2.88 V versus Fc^+/0^, which was assignable to W^IV‐III^ (Figure S1). This positive shift in the reduction potential of W^V‐IV^ aligned with the Fourier‐transform infrared (FT‐IR) spectroscopic observations, suggesting that Li^+^‐induced electron transfer lowers the energy barrier compared to the intrinsic reduction potential of W‐oxo bis(dithiolene) complexes. These results also indicate that the W^IV‐III^ reduction in bis(dithiolene) W‐oxo complexes, similar to proton‐coupled electron transfer processes [15], occurs readily due to Li^+^ cations, lowering the electron transfer energy. DFT calculations were employed to compare the thermodynamic energies of the binding of intermediates to Li^+^ ions across various oxidation states of the W complex to better understand this Li^+^‐induced electron transfer mechanism. These calculations can provide valuable insight into the charge/discharge mechanisms of the complexes.

**FIGURE 2:**
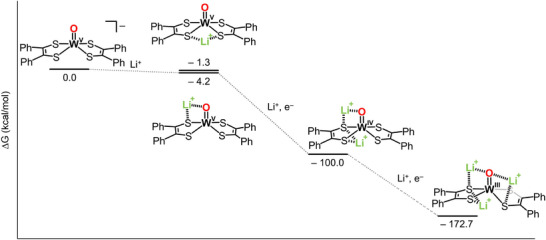
Energy diagram of Li^+^ binding and the electron transfer steps.

We compared the affinity of Li^+^ cations toward W oxidation states achievable within battery‐operating potential ranges (Figure 2). For (Et_4_N)[**1**], the interaction of W^V^═O with Li^+^ to form W^V^═O(µ_2_‐S,O:Li^+^) is thermodynamically favored, with a free energy decrease of −4.2 kcal/mol. Here, the Li^+^ cation interacts simultaneously with the W═O and dithiolene–S sites due to electron density localized at these sites. The W^V^═O(µ_2_‐S,O:Li^+^) interaction significantly lowers the energy barrier for electron transfer by 16.7 kcal/mol compared to the absence of Li^+^, consistent with electrochemical data showing a positive shift in the W^V‐IV^ reduction potential under LiPF_6_ conditions. Upon electron transfer, which forms a W^IV^ state, additional Li^+^ binding favors a W^IV^═O(µ_2_‐S,O:Li^+^)(µ_2_‐S,S’:Li^+^) by ∆G ═ −95.8 kcal/mol. Furthermore, additional Li^+^ cation binding facilitates subsequent electron transfer, promoting the reduction from W^IV^ to W^III^. The formation of W^III^═O(µ_2_‐S,O:Li^+^)_2_(µ_2_‐S,S’:Li^+^) is highly favorable, with a ∆G value of −72.7 kcal/mol. The atomic contribution to the redox processes can be assessed by analyzing the electron density distribution of the HOMO during the interaction between the complex and Li^+^. In the W(IV) state, most of the electron density was localized at the W center and dithiolene‐S sites, with negligible contribution from the O site. In contrast, upon reduction to the W(III) state, the contribution from the W─O site increased largely (Table S1). Additional electron transfer does not occur beyond this point within battery operational potentials. Consequently, interactions with Li^+^ cations effectively lower electron transfer energies, thereby facilitating the charging process. Moreover, the large HOMO‐LUMO energy gap in bis(dithiolene) W‐oxo complexes predisposes them to readily release electrons in a reverse direction, potentially aiding the discharge process [15].

**FIGURE 3:**
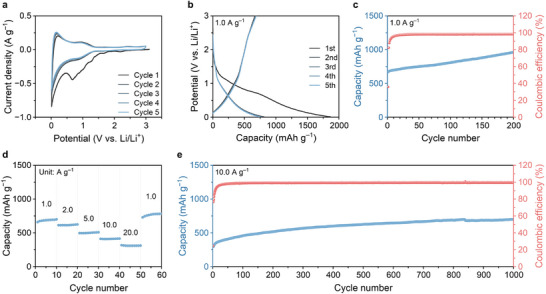
Electrochemical performance of the (Et_4_N)_2_[**1**] anode. (a) Initial five cyclic voltammograms at a scan rate of 0.1 mV s^−1^. (b) Initial five galvanostatic charge‐discharge voltage profiles at a current density of 1.0 A g^−1^. (c) Cycling performance at a current density of 1.0 A g^−1^. (d) Rate capability at various current densities. (e) Long‐term cycling performance at a high current density of 10.0 A g^−1^.

CR2032 coin‐type half‐cells were fabricated to investigate the electrochemical properties of the (Et_4_N)_2_[**1**] anode in LIBs. Figure 3a shows cyclic voltammograms of the (Et_4_N)_2_[**1**] anode recorded within a potential window of 0.01–3.0 V versus Li/Li^+^, at a scan rate of 0.1 mV s^−1^. During the first reduction process, the (Et_4_N)_2_[**1**] anode exhibited an irreversible cathodic peak at 0.66 V, which was attributed to the formation of a solid electrolyte interphase (SEI) layer resulting from the decomposition of electrolyte molecules on the electrode surface. After this initial irreversible cathodic sweep, the (Et_4_N)_2_[**1**] anode exhibited reversible redox activity associated with Li^+^ ion storage. A W^V‐IV^ redox couple was observed at around 1.80 V, while a W^IV‐III^ redox couple was observed around 0.85 V. These two‐step redox processes involve changes in the oxidation state of the tungsten metal centers in (Et_4_N)_2_[**1**], facilitating the insertion and extraction of two Li^+^ ions through electrochemical reactions.

Galvanostatic charge‐discharge cycling tests were carried out at various current densities to evaluate the practical cycling performance of the (Et_4_N)_2_[**1**] anode in LIBs. Figure 3b presents the galvanostatic charge–discharge voltage profiles of the (Et_4_N)_2_[**1**] anode over the initial five cycles at a current density of 1.0 A g^−1^. The cycling performance parameters were as follows (cycle number; charge capacity (mAh g^−1^), discharge capacity (mAh g^−1^), coulombic efficiency (CE, %)): (first; 669, 1866, 35.9), (second; 677, 812, 82.4), (third; 683, 769, 88.0), (fourth; 687, 752, 90.8), (fifth; 689, 742, 92.6). The (Et_4_N)_2_[**1**] anode exhibited slight voltage plateaus at approximately 0.9 and 1.75 V, which are consistent with the redox potentials observed in the cyclic voltammetry (CV) curves. As shown in Figure S2, these results were further confirmed by the dQ/dV curves derived from Figure 3b.

Figure 3c–e illustrates the galvanostatic charge‐discharge cycling performance of the (Et_4_N)_2_[**1**] anode at various current densities. At 1.0 A g^−1^, the (Et_4_N)_2_[**1**] anode exhibited a high reversible capacity of 963 mAh g^−1^ over 200 cycles, with an average CE value of 97.6% (Figure 3c). Figure 3d presents the rate capabilities of the (Et_4_N)_2_[**1**] anode, where the capacities were measured over 10 cycles at increasing current densities of 1.0, 2.0, 5.0, 10.0, and 20.0 A g^−1^. The 10th reversible capacity of the (Et_4_N)_2_[**1**] anode at each current density was 700, 625, 509, 415, and 311 mAh g^−1^. Upon returning to 1.0 A g^−1^, the reversible capacity of the (Et_4_N)_2_[**1**] anode recovered to 783 mAh g^−1^. Figure 3e displays the long‐term cycling performance at a high current density of 10.0 A g^−1^ over 1000 cycles. The first and 1000th reversible capacities of (Et_4_N)_2_[**1**] anode were 298 and 698 mAh g^−1^, with an average CE value of 99.0% over 1000 cycles.

**FIGURE 4:**
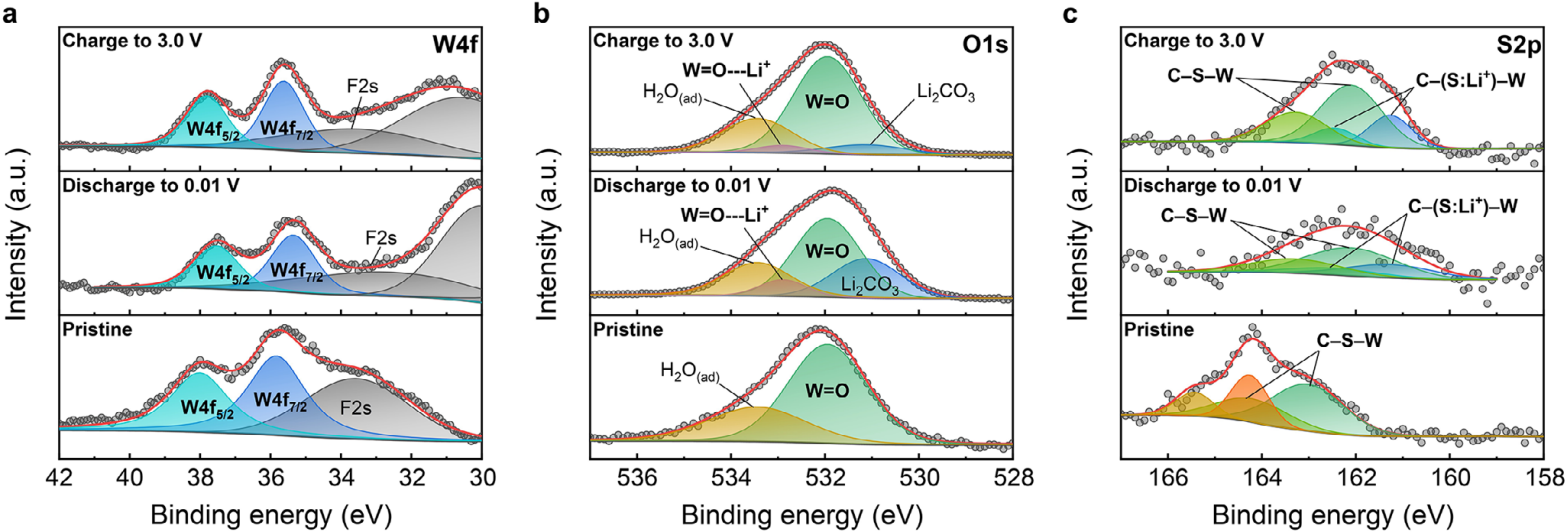
Ex situ XPS analysis of the (Et_4_N)_2_[**1**] anode in the pristine state, after full discharge to 0.01 V, and after recharge to 3.0 V; (a) W4f, (b) O1s, and (c) S2p XPS spectra.

Ex situ XPS analysis was conducted on electrode surfaces in the pristine state, after full discharge to 0.01 V, and after full recharge to 3.0 V to investigate the lithium‐ion storage mechanism of (Et_4_N)_2_[**1**] anode. The ex situ XPS analysis results are shown in Figure 4, where Figure 4a–c correspond to the W4f, O1s, and S2p regions, respectively. The detailed parameters used for the deconvolution of the XPS spectra are summarized in Tables S2–S4. The oxidation states of tungsten in the (Et_4_N)_2_[**1**] anode clearly changed during cycling (Figure 4a). W4f_5/2_ and W4f_7/2_ peaks were observed at 38.01 and 35.83 eV. After full discharge to 0.01 V, these peaks shifted to lower binding energies and shifted back to higher binding energies in the recharged state. In the O1s XPS spectra, the pristine (Et_4_N)_2_[**1**] anode exhibited a dominant peak corresponding to W═O species at 531.95 eV (Figure 4b). After discharge, the W═O peak remained dominant, and a new peak attributed to W═O─Li^+^ species emerged at 532.89 eV. The areal fraction of W═O─Li^+^ relative to the combined area of W═O and W═O─Li^+^ (relative W═O─Li^+^ ratio) was 13%. After recharging, the W═O─Li^+^ peak diminished, resulting in a decrease in the relative W═O─Li^+^ ratio to 5%. In addition, all O1s XPS spectra revealed peaks corresponding to adsorbed water, and Li_2_CO_3_ species, a well‐known component of the SEI layer, also appeared after the galvanostatic charge‐discharge cycling tests. In the S2p region, C–S–W peaks were observed at 164.20 eV (2p_1/2_) and 163.02 eV (2p_3/2_) (Figure 4c). After discharge, the peaks corresponding to C–S–W species decreased slightly, and new peaks appeared. The new peaks are presumed to originate from lithiation‐involved sulfur as C–(S:Li^+^)–W species, including W^V^═O(µ_2_‐S,O:Li^+^), W^IV^═O(µ_2_‐S,O:Li^+^)(µ_2_‐S,S’:Li^+^), and W^III^═O(µ_2_‐S,O:Li^+^)_2_(µ_2_‐S,S’:Li^+^) bonds. Then, the C–S–W peaks increased again and the C–(S:Li^+^)–W peaks were smaller in the recharged state. The areal fraction of C–(S:Li^+^)–W relative to the combined area of S2p_3/2_(C–S–W) and S2p_3/2_(C–(S:Li^+^)–W) (relative C–(S:Li^+^)–W ratio) decreased from 32% in the discharged state to 27% in the recharged state. The peaks corresponding to C–(S:Li^+^)–W remained after recharging because (Et_4_N)_2_[**1**] showed a thermodynamic preference for the formation of W^V^═O(µ_2_‐S,O:Li^+^) in the presence of LiPF_6_.

The W4f XPS spectra suggest that the coordinated tungsten center in (Et_4_N)_2_[**1**] was reduced during the discharge process and was reversibly oxidized during the charge process. As expected from the DFT‐based calculations, the O1s and S2p XPS spectra showed an increase and a subsequent decrease in the relative W═O─Li^+^ ratio and C–(S:Li^+^)–W ratio, respectively (Figure 2). Therefore, the results of the ex situ XPS analysis demonstrated that the redox processes of the (Et_4_N)_2_[**1**] anode in LIB systems originate from the redox reactions of tungsten metal centers, with their oxo and dithiolene ligands also participating in these redox processes.

**FIGURE 5:**
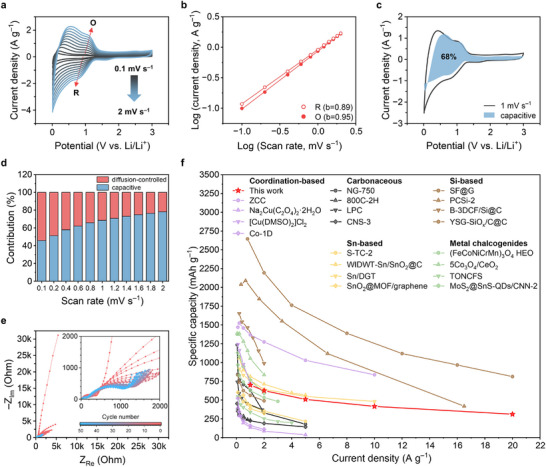
(a) CV curves of the (Et_4_N)_2_[**1**] anode at different scan rates. (b) Log‐log plots of peak current density versus scan rate for the (Et_4_N)_2_[**1**] anode, with corresponding *b* values. (c) Visualized capacitive current of the (Et_4_N)_2_[**1**] anode at 1 mV s^−1^. (d) Capacitive current contribution at different scan rates. (e) Nyquist plots of the (Et_4_N)_2_[**1**] anode during the first 50 cycles. (f) Cycling performance of promising LIB anode material candidates.

Various electrochemical analyses were conducted to further investigate the electrochemical properties of the (Et_4_N)_2_[**1**] anode. Figure 5a displays the CV curves obtained at various scan rates ranging from 0.1 to 2.0 mV s^−1^. Here, the resulting peak current densities provides insight into their electrochemical characteristics. The linear relationship between the logarithm of the peak current density and the logarithm of the scan rate is presented according to Equations (1) and (2) in the Experimental Methods section [20, 21], where the slope of each line corresponds to the *b* value (Figure 5b). The *b* value of the peaks was 0.89 (R) and 0.95 (O), indicating that redox reactions for the R and O peaks occur via capacitive processes. The capacitive contribution to the total electrochemical current was also analyzed using Dunn's method, which is not restricted to specific redox peaks (as described in Equation (3) in the Experimental Methods section) [22]. The (Et_4_N)_2_[**1**] anode exhibited dominant capacitive electrochemical characteristics, indicating sufficiently fast ion storage on its surfaces (Figure 5c,d).

Figure 5e presents Nyquist plots of the (Et_4_N)_2_[**1**] anode measured at the open‐circuit voltage before cycling and after each cycle up to 50 cycles. As cycling proceeded, the low‐frequency tail gradually diminished, and the mid‐frequency semicircle became smaller. Moreover, a new semicircle appeared in the high‐frequency region (Figure S3a), which is attributable to SEI formation. Because the EIS response evolved substantially upon cycling, it is difficult to consistently describe all spectra using a single fixed equivalent‐circuit model. Therefore, distribution of relaxation times (DRT) analysis was employed to track the evolution of interfacial resistive contributions of the (Et_4_N)_2_[**1**] anode in this work. This less model‐dependent approach represents the total impedance as an infinite series of parallel resistor‐capacitor (R||C) elements, each characterized by a relaxation time constant (τ) [23]. A detailed discussion of the evolution of the DRT peaks (P1–P5) is described below Figure S3 in the Supporting Information. The DRT analysis (Figure S3b–d) reveals that the relaxation time and intensity of P3 and P4, as well as the overall polarization resistance (R_p_), decreased markedly within the first 10 cycles, indicating an activation process. This behavior may reflect the electrolyte wetting and the formation and reorganization of interfacial resistive layers such as SEI, leading to reduced interfacial polarization and improved charge transfer kinetics. These impedance evolutions are consistent with the improved Coulombic efficiency and increased reversible capacity during the first 10 cycles, as shown in Figure 3.

Notably, the measured reversible capacity substantially exceeds the theoretical capacity of the (Et_4_N)_2_[**1**] (57 mAh g^−1^, n = 2), implying additional charge storage contributions beyond the nominal two‐electron redox process. To gain further insight, the (Et_4_N)_2_[**1**] anode at the fully discharged state after the first and 50 cycles was analyzed using transmission electron microscopy (TEM) (Figure S4). Figure S4a–c show lattice fringes corresponding to the (110) plane of metallic Li, and a higher density of metallic Li nanoclusters is observed after 50 cycles compared with the first discharge (Figure S4c) [7]. This trend is in line with the progressive decrease of the P3 and P4 contributions in the DRT spectra, suggesting that the interfacial environment becomes more electrochemically accessible upon cycling. Such interfacial evolution may facilitate metallic Li nanoclusters formation and correlate with the gradual capacity increase.

Figure 5f visualizes the comparison between the cycling performances of the (Et_4_N)_2_[**1**] anode in this work and those of various other recently reported promising anode material candidates [24, 25, 26, 27, 28, 29, 30, 31, 32, 33, 34, 35, 36, 37, 38, 39]. The (Et_4_N)_2_[**1**] anode in this work demonstrated outstanding specific capacities and rate performances at high current densities of up to 20 A g^−1^. Notably, its cycling performance was superior to that of recently reported tin‐based [24, 25, 26, 27], metal‐chalcogenide‐based [28, 29, 30, 31], and carbonaceous anode materials [32, 33, 34, 35], as well as coordination‐based anode materials [7, 8, 9, 10]. Although the (Et_4_N)_2_[**1**] anode delivered relatively low capacities compared to silicon‐based materials [36, 37, 38, 39], it showed strength in terms of rate capability. The superb cycling performance of the (Et_4_N)_2_[**1**] anode is assumed to stem from its well‐combined electrochemical kinetics, arising from excellent ionic and electronic conductivities and predominant capacitive electrochemical behaviors.

## 3 Conclusions

In this work, (Et_4_N)_2_[WO(pdt)_2_], a redox‐active tungsten mono‐oxo bis(dithiolene) complex, was successfully applied as a fast‐rechargeable novel anode material for LIBs. Its lithium‐ion storage capability was demonstrated using FT‐IR spectroscopy and CV measurements in the presence of LiPF_6_. The LIB performance of the W complex anode was evaluated using CV and the galvanostatic charge‐discharge cycling tests. The (Et_4_N)_2_[WO(pdt)_2_] anode exhibited reversible redox behaviors and an excellent reversible capacity of 963 mAh g^−1^ at 1.0 A g^−1^, as well as an outstanding rate performance with a reversible capacity of 698 mAh g^−1^ at a high current density of 10.0 A g^−1^ after 1000 cycles. These fast‐rechargeable properties of the (Et_4_N)_2_[WO(pdt)_2_] anode were a result of its predominant capacitive behaviors. Ex situ XPS analysis and DFT‐based simulations elucidated the lithium‐ion storage mechanism of (Et_4_N)_2_[WO(pdt)_2_] and showed that the reversible redox reactions occur not only through changes in the oxidation state of the tungsten metal center but also involve the O_oxo_ and S_dithiolene_ sites during lithiation and delithiation. To the best of our knowledge, this is the first report of the application of a redox‐active dithiolene‐based molecular coordination complex as an electrode material for LIBs. It is hoped that the study findings will contribute to the development of tungsten‐based complexes and other metal dithiolene complexes as next‐generation anode materials for LIBs.

## 4 Experimental Methods

### 4.1 Materials

All complexes were synthesized under an N_2_ or Ar atmosphere using glovebox and Schlenk techniques. Solvents were purified using a solvent purification system (Vigor) and stored over 4 Å molecular sieves until used. Tetrabutylammonium hexafluorophosphate (Acros, ≥ 98%) was recrystallized from ethanol. Tungsten hexacarbonyl (Sigma‐Aldrich, 97%), nickel chloride hexahydrate (Alfa Aesar, 99.95%), phosphorus pentasulfide (Acros, +98%), benzoin (Acros, 98%), tetraethylammonium hydroxide (Alfa Aesar, 25 wt.% solution in methanol), lithium hexafluorophosphate (TCI, 97%), sodium mercury amalgam (Acros, ca. 20% sodium), 1‐methyl‐2‐pyrrolidone (NMP, Sigma‐Aldrich, ≥99.0%), and poly(vinylidene fluoride) (PVDF, Sigma‐Aldrich, average Mw ∼534,000 by GPC, powder) were used as received. Lithium metal foil was purchased from MTI Corp. Conductive carbon black (Super P), polypropylene (PP) membrane (Celgard 3501), 18 µm‐thick copper foil, 1 M LiPF_6_ in ethylene carbonate (EC) and dimethyl carbonate (DMC) (1:1 v/v), and the CR2032 coin‐type cell assembly packages were purchased from Wellcos Co.

### 4.2 Characterization

^1^H‐NMR (400 MHz) spectra were measured on a JEOL NMR spectrometer (JNM‐ECS400). All NMR samples were prepared in screw‐cap NMR tubes inside an Ar‐purged glove box. Fourier‐transform infrared (FT‐IR) spectra were collected using an FT‐IR spectrometer (ALPHA II) equipped with a universal sampling module (A230/D, QuickSnap). All complexes were drop‐cast onto the KBr round crystal window (Sigma‐Aldrich, 25 mm in diameter × 4 mm thickness) in solution, and the solvent was evaporated to adsorb to the KBr window. Mineral oil was applied to the sample adsorbed on the KBr window, and another KBr window was placed on top of it to prevent contamination. X‐ray photoelectron spectroscopy (XPS) was performed using a Thermo VG Scientific Sigma Probe spectrometer (monochromatic Al Ka radiation, hν = 1486.6 eV). Transmission electron microscopy (TEM) measurement was performed on a FEI TECNAI TF30ST at an acceleration voltage of 300 kV. The cyclic voltammetry (CV) experiments in Figure S1 were performed using an INTERFACE 1010 E Potentiostat/Galvanostat/ZRA. The CV studies were conducted in a three‐electrode cell equipped with a glassy carbon disk (3.0 mm diameter) working electrode, a platinum wire counter electrode, and an Ag/AgNO_3_ (0.01 M)/CH_3_CN non‐aqueous reference electrode (also containing 0.1 M nBu_4_NPF_6_), separated from the solution by a porous CoralPor tip. The working electrode was polished prior to each experiment using a 0.05 µm alumina polishing agent on a pad. The electrolyte was 0.1 M tetrabutylammonium hexafluorophosphate (nBu_4_NPF_6_) in 10 mL of freshly prepared CH_3_CN and saturated with Ar or N_2_. At the conclusion of each experiment, the potentials were referenced against ferrocene/ferrocenium (Fc/Fc^+^) as the external standard.

### 4.3 Synthesis of (Et_4_N)_2_[**1**]

A solution of 300 mg (0.414 mmol) of [W(CO)_2_(S_2_C_2_Ph_2_)_2_] in 50 mL of THF was treated with 1.7 mL of a 25% solution (w/w) of Et_4_NOH in methanol (2.55 mmol). Solvent was removed in vacuo and washed with THF (3 × 10 mL). Orange residue was redissolved in acetonitrile and recrystallized from acetonitrile/ether. The product was isolated as 316.9 mg (81%) of an orange‐red crystalline solid. IR (KBr): ν_WO_ 886 cm^−1^ [19].

### 4.4 Synthesis of (Et_4_N)[**1**]

A stirred suspension of 51 mg (54 µmol) of (Et_4_N)_2_[WO(S_2_C_2_Ph_2_)_2_] in 2 mL of acetonitrile was treated dropwise with 6.8 mg (27 µmol) of iodine in 1 mL of THF. Solvent was removed in vacuo, and the dark residue was redissolved in THF. The solution was filtered to remove Et_4_NI. Ether was diffused into the filtrate, causing the product to separate as 34 mg (78%) of a purple crystalline solid. IR (KBr): ν_WO_ 940 cm^−1^ [19].

### 4.5 Electrochemical Tests for LIBs

(Et_4_N)_2_[**1**] anodes were prepared by mixing 30 mg of (Et_4_N)_2_[**1**] powder (active material), 60 mg of conductive carbon (Super P), and 10 mg of PVDF binder in NMP solvent using an agate mortar. The resulting homogeneous black slurry was cast onto 18 µm‐thick Cu foil using a doctor blade. The coated electrodes were dried at 80°C in air for 1 h, followed by further drying under vacuum for 3 h. The dried electrodes were then punched into 10 mm diameter discs to serve as working electrodes. Li foil was used as a counter/reference electrode, Celgard 3501 PP membrane was used as the separator, and 1 M LiPF_6_ was used in a 1:1 (v/v) mixture of EC and DMC as the electrolyte. All CR2032 coin‐type lithium half‐cells were assembled in an argon‐filled glove box (O_2_ and H_2_O < 0.1 ppm). CV studies for Li‐ion half‐cells and galvanostatic charge‐discharge cycling tests were performed using a WonAtech WBCS3000S in a potential window of 0.01–3.0 V versus Li/Li^+^. Electrochemical impedance spectroscopy (EIS) was carried out over a frequency range of 1 MHz to 10 mHz using a WonAtech ZIVE SP1.

The electrochemical kinetics of the Li‐ion storage process were further analyzed based on the response of the peak current densities to the redox peaks, as described by the following equations [20, 21],

(1) $$i=av^{b}$$

(2) logi=loga+blogv

where *i* and *v* represent the peak current and the scan rate, respectively. A *b* value near 0.5 indicates that the electrochemical process is predominantly diffusion‐controlled, while a *b* value close to 1 suggests a surface‐controlled (capacitive) reaction.

Equation (3) was used to elucidate the electrochemical behavior by distinguishing between diffusion‐ and surface‐controlled contributions to the total electrochemical current, according to Dunn's method [22]:

(3) $$i_{t}=k_{1}v+k_{2}v^{0.5}$$

Here, *i_t_*, *k*_1_ *v*, and *k*_2_ *v* ^0.5^ represent the total current, the capacitive‐controlled current, and the diffusion‐controlled current, respectively.

### 4.6 Computational Details

The geometries were optimized using the starting structure taken from the single‐crystal structure of (Et_4_N)_2_[**1**]. Geometric optimizations and frequency calculations were performed with the unrestricted B3LYP functional, def2‐SVP basis set, and effective core potentials using Orca 5.01 software [40, 41, 42, 43, 44, 45]. For faster calculations, the RI‐J approximation was used, with the general auxiliary def2/J basis set by Weigend, as implemented in ORCA [46]. Solvation free energies in the 1:1 mixture of ethylene carbonate and dimethyl carbonate were calculated by the polarizable continuum model (C‐PCM) using Bondi atomic radii with a dielectric constant of 22.94 [47, 48, 49, 50]. The single‐point energy of the obtained optimized geometries was calculated using the unrestricted B3LYP functional, def2‐TZVPPD basis set [45], effective core potentials, dispersion correction, and the auxiliary def2/J basis set [46] for all atoms. Frequency calculations were performed on all stationary points. Gibbs free energies (ΔG), including zero‐point vibrational energies (ΔZ_0_), vibrational energies at 298 K (ΔE_thermal_), entropy at 298 K (−TΔS), and dispersion (ΔDisp) were calculated [51].

## Funding

National Research Foundation of Korea. Grant Numbers: RS‐2024‐00411257, RS‐2024‐00412170, RS‐2021‐NR060081, RS‐2023‐00301974.

## Conflicts of Interest

The authors declare no conflict of interest.

## Supporting information




**Supporting File**: advs75010‐sup‐0001‐SuppMat.docx.

## Data Availability

The data that support the findings of this study are available in the supplementary material of this article.
