# Supplementary material for: Redox‐Active Tungsten Mono‐Oxo Bis(dithiolene) Complex: A Fast‐Rechargeable Anode for High‐Capacity Lithium‐Ion Batteries
Source: Adv Sci (Weinh). 2026 Mar 25;13(32):e75010. doi: 10.1002/advs.75010 (PMC13252656; doi:10.1002/advs.75010)
Supplement: Supplementary file 1 — Supporting File: advs75010‐sup‐0001‐SuppMat.docx. [file ADVS-13-e75010-s001.docx]

Supporting Information

Redox-Active Tungsten Mono-Oxo Bis(dithiolene) Complex: A Fast-Rechargeable Anode for High-Capacity Lithium-Ion Batteries

Honggyu Seong^a,b,†^, Jaeheon Lee^c,d,†^, Jae Hyun Park^e^, Woonghee Lee^f^, Junhyeok Seo^c,d,^* and Jaewon Choi^a,b,^*


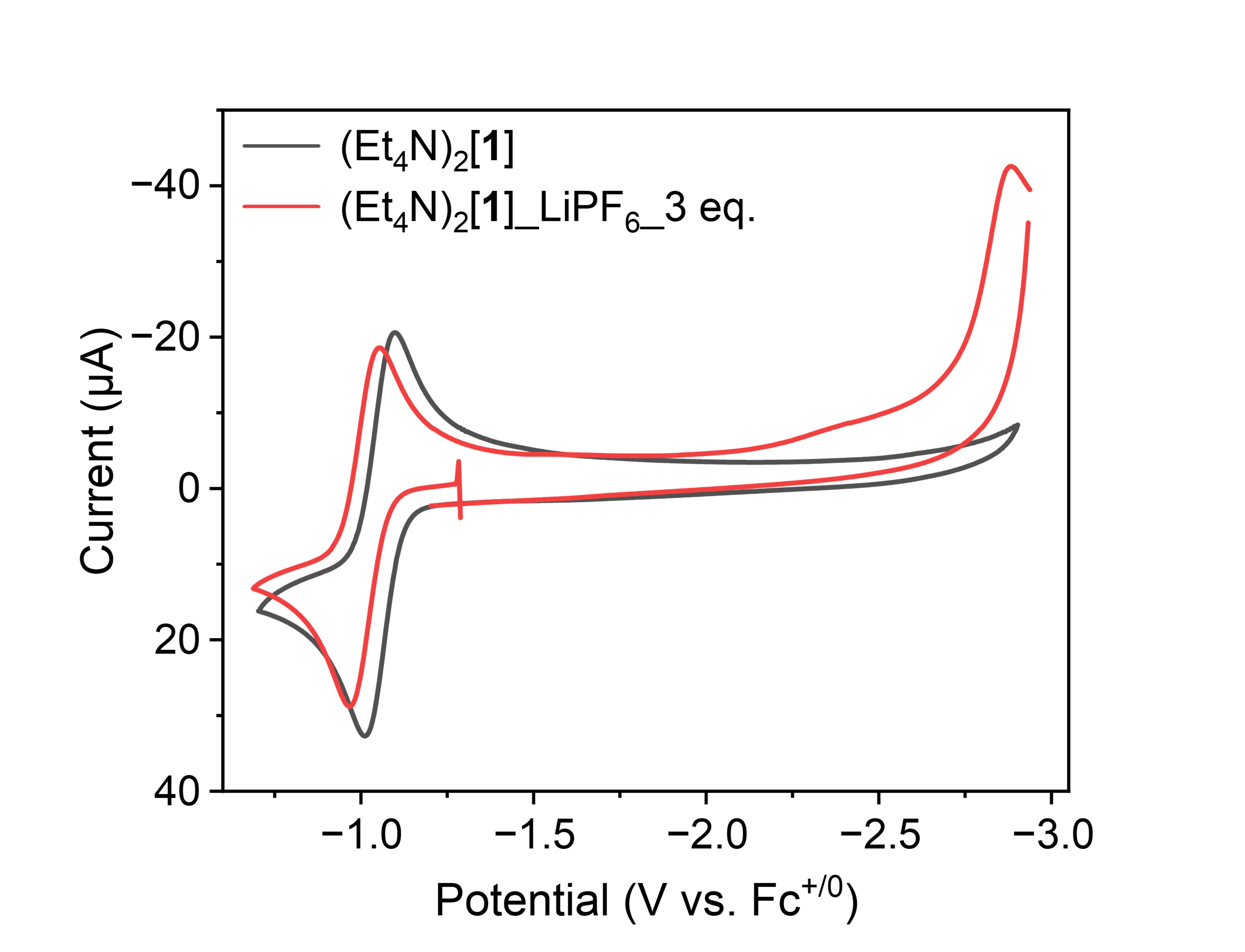


**Figure S1.** Cyclic voltammogram of (Et_4_N)_2_[**1**] with 3 equivalents of LiPF_6_.


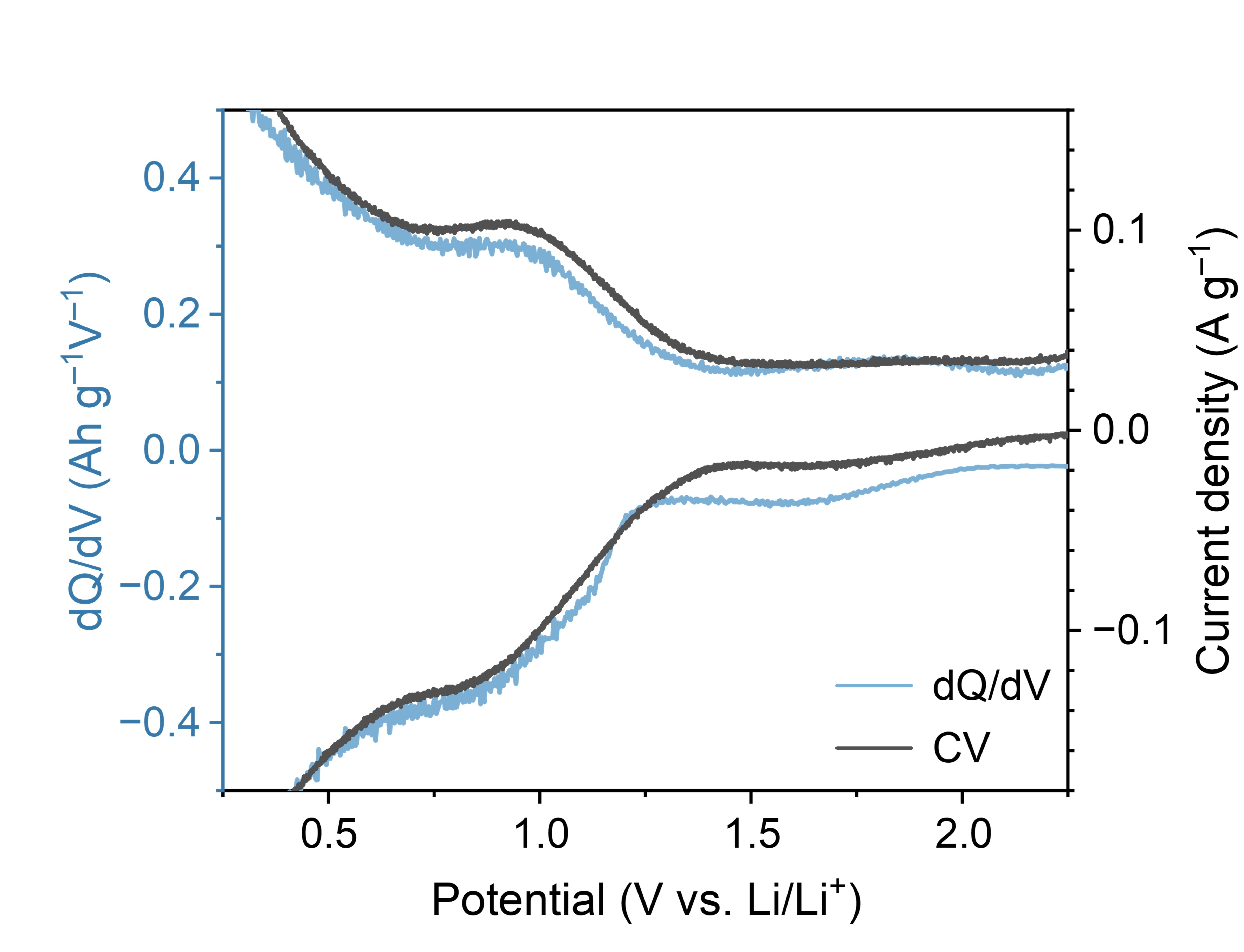


**Figure S2.** dQ/dV curve of the (Et_4_N)_2_[**1**] anode, compared with its corresponding CV curve.


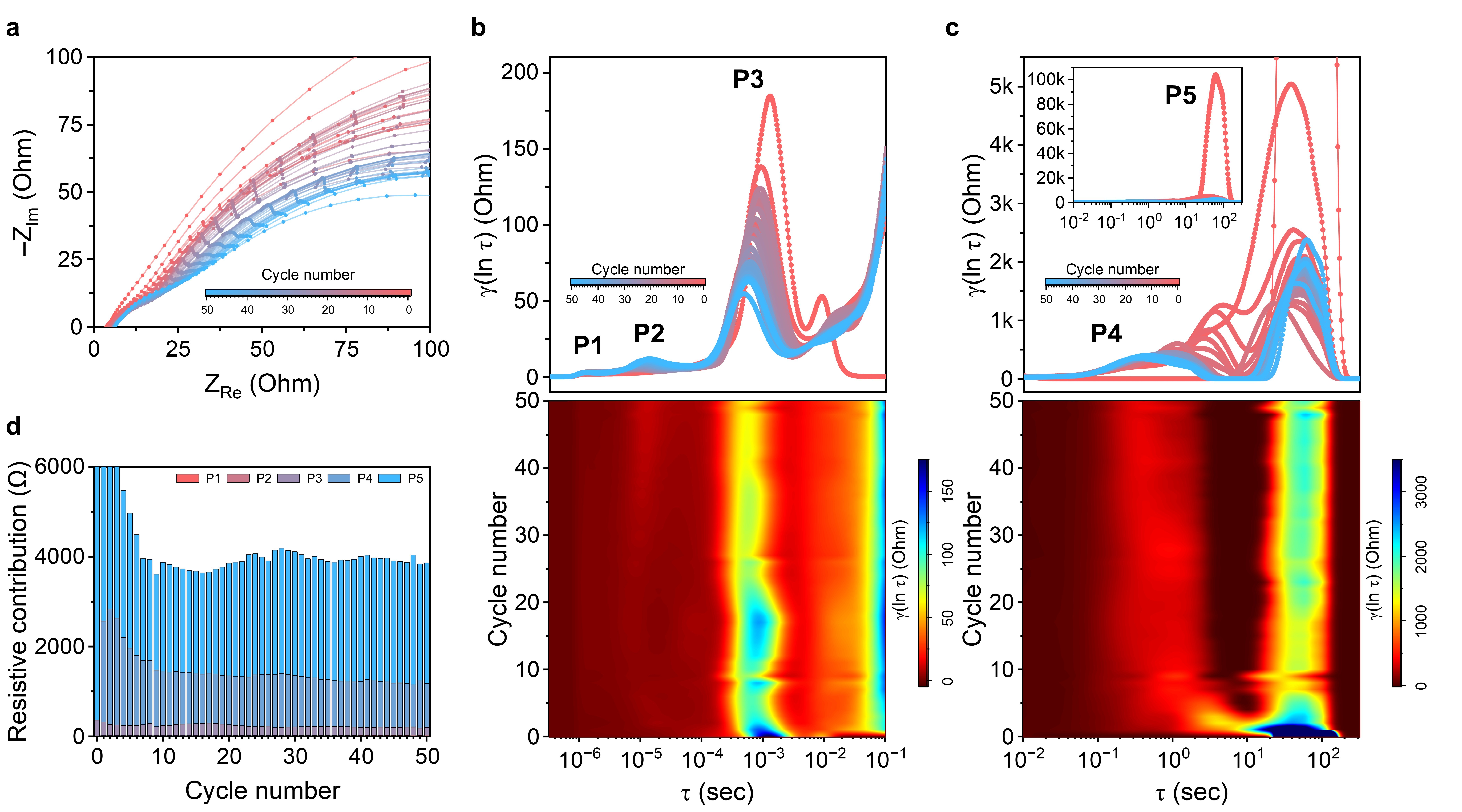


**Figure S3.** (a) Zoomed-in Nyquist plots in the high-frequency region. (b, c) DRT spectra derived from EIS measured at OCV after each recharge to 3.0 V over the first 50 cycles. (d) Resistive contributions of the individual processes across cycles.

Figure S3b and S3c shows the evolution of DRT spectra over 50 cycles, where five DRT peaks (P1–P5) were observed at distinct relaxation times, indicating multiple electrochemical processes. In the short relaxation time range (Figure S3b), P1, P2, and P3 were located around 1.2 × 10^–6^, 1.6 × 10^–5^, and 1.3 × 10^–3^ s, respectively. P1 is a minor peak that is already present before cycling and is assigned to the ohmic resistance contribution. P2 is also a small peak but emerges only after cycling. Its evolution is consistent with the additional high frequency semicircle observed in Figure S3a, suggesting that P2 is associated with the SEI formation. Notably, P3, the dominant peak in this relaxation time range, decreased upon cycling and shifted toward shorter relaxation times to 6.70 × 10^–4^ s within the first 10 cycles, and further to 5.60 × 10^–4^ s over 50 cycles. These trends indicate that P3 is associated with the main interfacial charge transfer process with its improvement during cycling, consistent with the reduced mid frequency semicircle in the Nyquist plots.

In the long relaxation time range (Figure S3c), the DRT spectrum was initially dominated by a large peak (P5) centered at 62.97 s at open-circuit voltage before cycling. During the initial 10 cycles, this low-frequency response became more clearly resolved into P5 and an additional contribution (P4) around 0.6 s. Then, P4 steadily redistributed and decreased upon further cycling test, suggesting the stabilization of an initially heterogeneous interfacial state.

For the quantitative interpretation, polarization resistance (R_p_) was obtained by integrating the DRT spectrum over ln τ. Figure S3d depicts R_p_ and the integrated resistive contributions corresponds to P1–P5 ranges (denoted as R1–R5, respectively) over cycle number. R_p_ decreased from 120 kΩ before cycling to 3.88 kΩ within the initial 10 cycles and then remained nearly constant (3.86 kΩ over 50 cycles). After 50 cycles, R1–R5 were 3, 24, 178, 971, 2686 Ω, respectively, and their average fractional contributions from cycles 10–`50 were 0.07, 0.45, 5.32, 27.82, 66.34%, respectively. These results indicate that the low-frequency polarization (P4 and P5), such as distributed interfacial relaxation and mass transport related limitations, is substantial fraction of the total electrochemical impedance of the (Et_4_N)_2_[**1**] anode.





**Figure S4.** TEM images of the (Et_4_N)_2_[**1**] anode at the fully discharged state after (a, b) the 50th cycle (different magnifications) and (c) the first cycle.

**
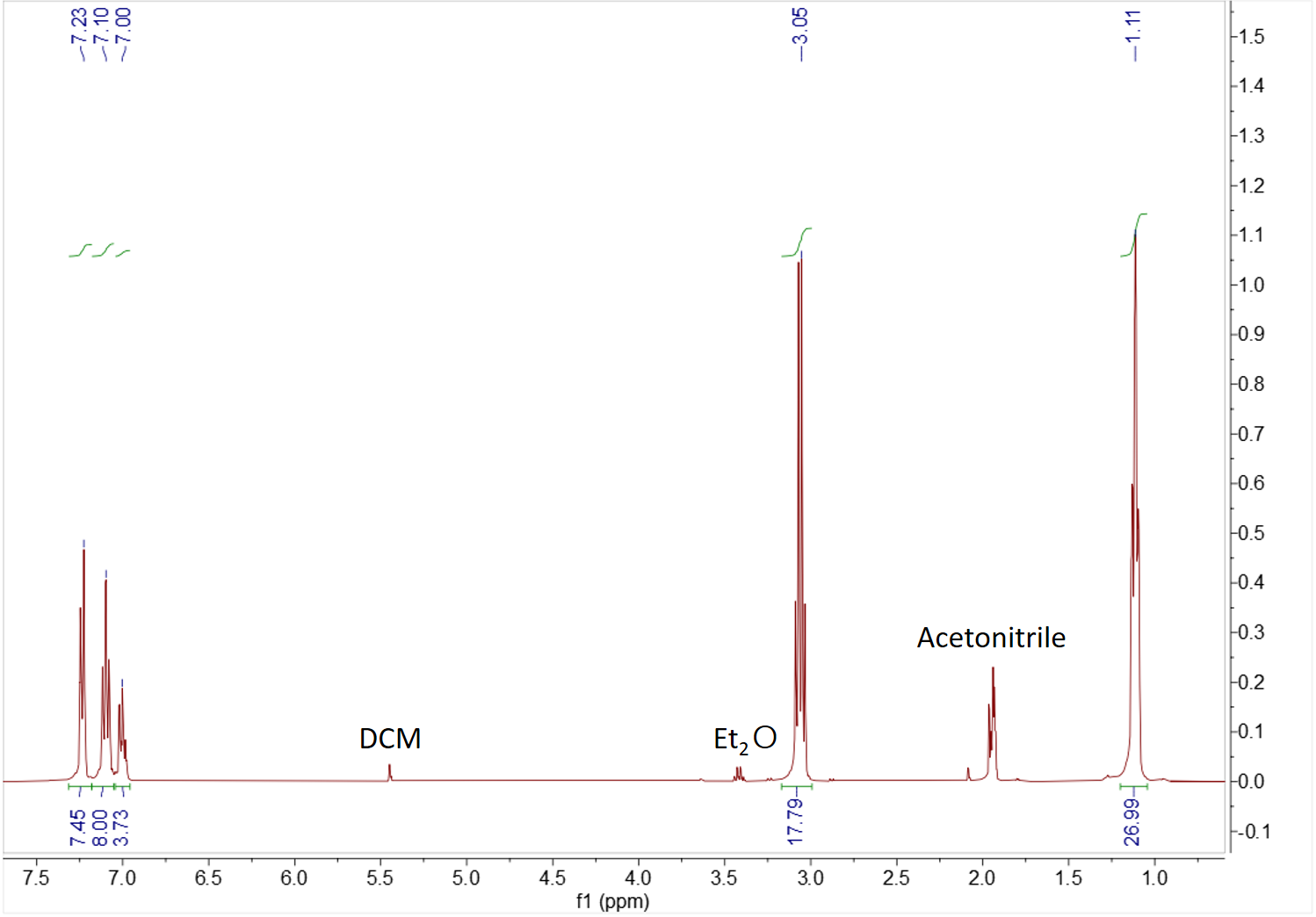
**

**Figure S5.** ^1^H NMR data of (Et_4_N)_2_[WO(S_2_C_2_Ph_2_)_2_]

**Table S1.** Atomic contribution for HOMO of W^IV^=O(μ_2_-S,O:Li^+^) and W^III^=O(μ_2_-S,O:Li^+^)(μ_2_-S,S’:Li^+^)

|  | W metal center | Dithiolene S_4_ | Oxygen atom |
| --- | --- | --- | --- |
| W^IV^=O(μ_2_-S,O:Li^+^) | 60% | 20% | 1% |
| W^III^=O(μ_2_-S,O:Li^+^)(μ_2_-S,S’:Li^+^) | 54% | 18% | 16% |

**Table S2.** Deconvolution parameters of the W4f XPS spectra.

| Charge state | Peak | Position (eV) | FWHM (eV) | Area (a.u.) |
| --- | --- | --- | --- | --- |
| Pristine | F2s | 33.56 | 3.45 | 461.40 |
|  | W4f_7/2_ | 35.83 | 1.94 | 452.70 |
|  | W4f_5/2_ | 38.01 | 1.94 | 339.53 |
| Discharge  to 0.01 V | F2s | 32.69 | 5.84 | 359.65 |
|  | W4f_7/2_ | 35.35 | 1.50 | 251.22 |
|  | W4f_5/2_ | 37.53 | 1.50 | 188.42 |
|  | unknown | 29.98 | 2.75 | 680.65 |
|  | unknown | 25.38 | 4.71 | 884.35 |
| Charge  to 3.0 V | F2s | 33.56 | 4.91 | 452.26 |
|  | W4f_7/2_ | 35.62 | 1.37 | 415.33 |
|  | W4f_5/2_ | 37.80 | 1.37 | 311.50 |
|  | unknown | 30.55 | 3.94 | 914.70 |
|  | unknown | 25.23 | 4.25 | 719.36 |

**Table S3.** Deconvolution parameters of the O1s XPS spectra.

| Charge state | Peak | Position (eV) | FWHM (eV) | Area (a.u.) |
| --- | --- | --- | --- | --- |
| Pristine | W=O | 531.95 | 1.97 | 4166.67 |
|  | H_2_O | 533.44 | 2.47 | 1906.08 |
| Discharge  to 0.01 V | Li_2_CO_3_ | 531.12 | 1.69 | 7504.84 |
|  | W=O | 531.95 | 1.65 | 14682.68 |
|  | W=O---Li^+^ | 532.89 | 1.15 | 2133.43 |
|  | H_2_O | 533.44 | 1.69 | 6883.11 |
| Charge  to 3.0 V | Li_2_CO_3_ | 531.12 | 1.95 | 2417.54 |
|  | W=O | 531.95 | 1.69 | 21026.98 |
|  | W=O---Li^+^ | 532.89 | 1.25 | 1166.04 |
|  | H_2_O | 533.44 | 1.74 | 7466.79 |

**Table S4.** Deconvolution parameters of the S2p XPS spectra.

| Charge state | Peak | Position (eV) | FWHM (eV) | Area (a.u.) |
| --- | --- | --- | --- | --- |
| Pristine | C–S–W (2p_3/2_) | 163.02 | 1.80 | 170.46 |
|  | C–S–W (2p_1/2_) | 164.20 | 1.80 | 85.23 |
|  | unknown (2p_3/2_) | 164.26 | 0.94 | 88.48 |
|  | unknown (2p_1/2_) | 165.44 | 0.94 | 44.24 |
| Discharge  to 0.01 V | C–(S:Li^+^)–W (2p_3/2_) | 161.26 | 2.20 | 38.29 |
|  | C–(S:Li^+^)–W (2p_1/2_) | 162.44 | 2.20 | 19.15 |
|  | C–S–W (2p_3/2_) | 162.10 | 2.25 | 82.84 |
|  | C–S–W (2p_1/2_) | 163.28 | 2.25 | 41.42 |
| Charge  to 3.0 V | C–(S:Li^+^)–W (2p_3/2_) | 161.26 | 1.04 | 82.44 |
|  | C–(S:Li^+^)–W (2p_1/2_) | 162.44 | 1.04 | 41.22 |
|  | C–S–W (2p_3/2_) | 162.10 | 1.52 | 223.73 |
|  | C–S–W (2p_1/2_) | 163.28 | 1.52 | 111.87 |
